# Six-fold C–H borylation of hexa-*peri*-hexabenzocoronene

**DOI:** 10.3762/bjoc.16.37

**Published:** 2020-03-13

**Authors:** Mai Nagase, Kenta Kato, Akiko Yagi, Yasutomo Segawa, Kenichiro Itami

**Affiliations:** 1 Graduate School of Science, Nagoya University, Chikusa, Nagoya, 464-8602, Japan; 2 JST-ERATO, Itami Molecular Nanocarbon Project, Nagoya, 464-8602, Japan; 3 Institute of Transformative Bio-Molecules (WPI-ITbM), Nagoya University, Chikusa, Nagoya, 464-8602, Japan

**Keywords:** C–H borylation, hexa-*peri*-hexabenzocoronene, iridium catalyst, X-ray crystallography

## Abstract

Hexa-*peri*-hexabenzocoronene (HBC) is known to be a poorly soluble polycyclic aromatic hydrocarbon for which direct functionalization methods have been very limited. Herein, the synthesis of hexaborylated HBC from unsubstituted HBC is described. Iridium-catalyzed six-fold C–H borylation of HBC was successfully achieved by screening solvents. The crystal structure of hexaborylated HBC was confirmed via X-ray crystallography. Optoelectronic properties of the thus-obtained hexaborylated HBC were analyzed with the support of density functional theory calculations. The spectra revealed a bathochromic shift of absorption bands compared with unsubstituted HBC under the effect of the σ-donation of boryl groups.

## Introduction

Polycyclic aromatic hydrocarbons (PAHs) are beneficial chemical compounds as semiconductors and luminescent materials [1–3]. Direct functionalizations of PAHs are extensively studied because physical and electronic properties can be tuned by their peripheral substituents [4–6]. For instance, the introduction of electron-donating or -withdrawing groups alters electronic properties, as well as the solubility of PAHs increases by introducing long alkyl chains or bulky substituents.

In general, it is indispensable to directly functionalize large molecular weight PAHs [7]. As the molecular weight of planar PAHs increases, solubility in common organic solvents greatly decreases due to the π–π interactions. Hexa-*peri*-hexabenzocoronene (HBC) is a notable compound investigated in the fields from synthetic chemistry to astrophysics among PAHs [4,8–10], and also known as a poorly soluble PAH [11]. Against the context of difficulty in functionalizing poorly soluble compounds, functionalized HBC is typically synthesized by dehydrogenative cyclization, which is called Scholl reaction, of precursors having solubilizing substituents [12–13]. However, the Scholl reaction sometimes causes unexpected rearrangement such as 1,2-aryl shift, and produces undesired cyclic compounds [14]. To design and synthesize a variety of HBC derivatives, methods for regioselective functionalization of HBC are in strong demand.

**Figure 1:**
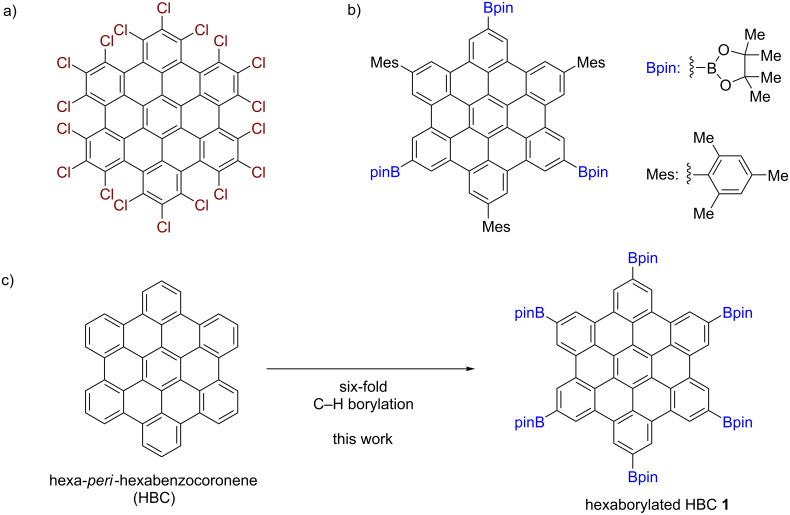
C–H functionalization of HBCs. (a) Perchlorinated HBC. (b) Borylated HBC substituted by 2,4,6-trimethylphenyl (Mes) groups. (c) Six-fold C–H borylation of HBC (this work).

As for functional groups on the HBC core, the introduction of halogen and boryl groups is an effective way to enable further functionalization [15]. Perchlorination was reported to functionalize HBC directly (Figure 1a) [16], and perchlorinated HBC was utilized for the formation of multiply functionalized HBC derivatives [17–18]. Other than perchlorination, hexaiodinated HBC was employed in subsequent reactions to introduce substituents [19]. However, there is no report on the hexaborylation of unfunctionalized HBC, although Shinokubo and co-workers reported the di- and triborylation of HBCs substituted by 2,4,6-trimethylphenyl (Mes) groups as solubilizing groups (Figure 1b) [20]. Since boryl groups can be converted into various functional groups [15], hexaborylated HBC is expected to be a platform for the synthesis of diverse functionalized HBCs.

Herein, we report the six-fold C–H borylation of unfunctionalized HBC (Figure 1c). By screening solvents, we have established suitable reaction conditions for the synthesis of hexaborylated HBC **1**. The structure of thus-obtained **1** was confirmed by X-ray crystallography, and the electronic effects of the boryl groups were investigated through optoelectronic measurements and density functional theory (DFT) calculations.

## Results and Discussion

**Figure 2:**
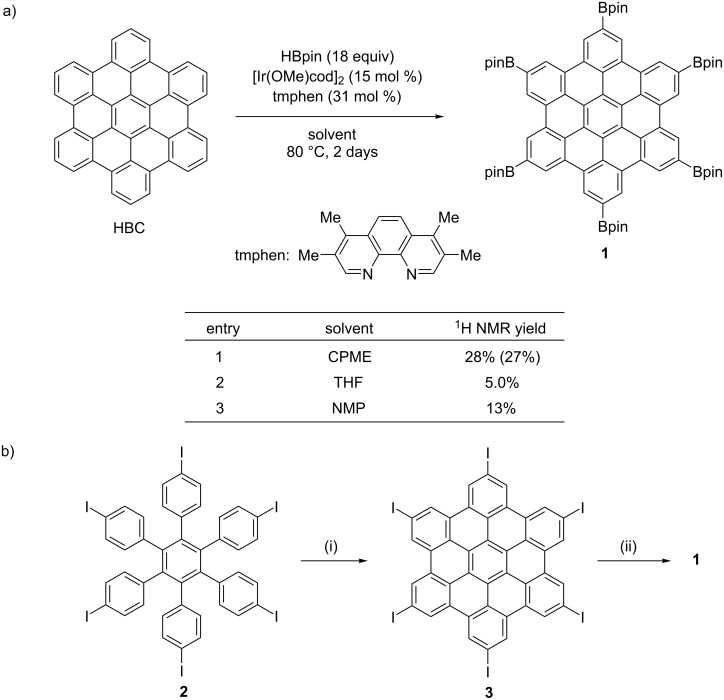
Synthesis of hexaborylated HBC **1.** (a) Solvent screening of six-fold C–H borylation of unsubstituted HBC. 2,3,4-Trimethoxybenzaldehyde was used as an internal standard to estimate the yield of **1**. The yield in parentheses is the isolated yield of **1**. (b) Stepwise synthesis of **1** via Miyaura–Ishiyama borylation. Reaction conditions: (i) FeCl_3_ (32 equiv), CH_3_NO_2_/CH_2_Cl_2_, rt, 21 h; (ii) B_2_pin_2_ (10 equiv), Pd(dppf)Cl_2_ (50 mol %), KOAc (6.2 equiv), toluene/DMF (1:1), 80 °C, 18 h.

We have examined the conditions for C–H borylation of unsubstituted HBC. HBC was pulverized by a ball mill prior to use. First, we attempted the iridium catalyst and reagents that we have reported as the suitable C–H borylation conditions for warped nanographene: [Ir(OMe)cod]_2_, 3,4,7,8-tetramethyl-1,10-phenanthroline (tmphen), HBpin (Bpin: 4,4,5,5-tetramethyl-1,3,2-dioxaborolan-2-yl) and cyclopentyl methyl ether (CPME) [21]. With these conditions at 80 °C for 2 days, **1** was afforded in 27% isolated yield (Figure 2a, entry 1), albeit the solubility of HBC in CPME was quite low. As a common solvent for C–H borylation, THF was used at 80 °C in a sealed tube to give 5.0% yield (Figure 2a, entry 2). When applying the same conditions in *N*-methylpyrrolidone (NMP) aiming to improve the solubility of HBC [11], **1** was obtained in 13% yield (Figure 2a, entry 3). The obtained hexaborylated HBC **1** can be dissolved in various solvents such as THF, ethyl acetate, diethyl ether, ethanol, CH_2_Cl_2_ and CHCl_3_.

To compare the efficiency of the current method, the stepwise synthesis of **1** was also examined. The synthesis of hexaiodoinated HBC **3** was conducted through Scholl reaction according to a literature procedure [19]. The subsequent Miyaura–Ishiyama borylation [22] was carried out with B_2_pin_2_ in the presence of Pd(dppf)Cl_2_ (50 mol %) and KOAc (6.2 equiv) in toluene/DMF (1:1) at 80 °C for 18 h to afford **1** in 0.8% yield based on **2** (Figure 2b). This result suggests the usefulness of the direct borylation of unsubstituted HBC although the yield of the Miyaura–Ishiyama borylation step is possibly increased by further conditions screening. The thus-obtained **1** was identical to the product from the C–H borylation of HBC according to ^1^H NMR spectroscopy, and the ^13^C NMR and HRMS by MALDI–TOF MS results also supported the identification.

**Figure 3:**
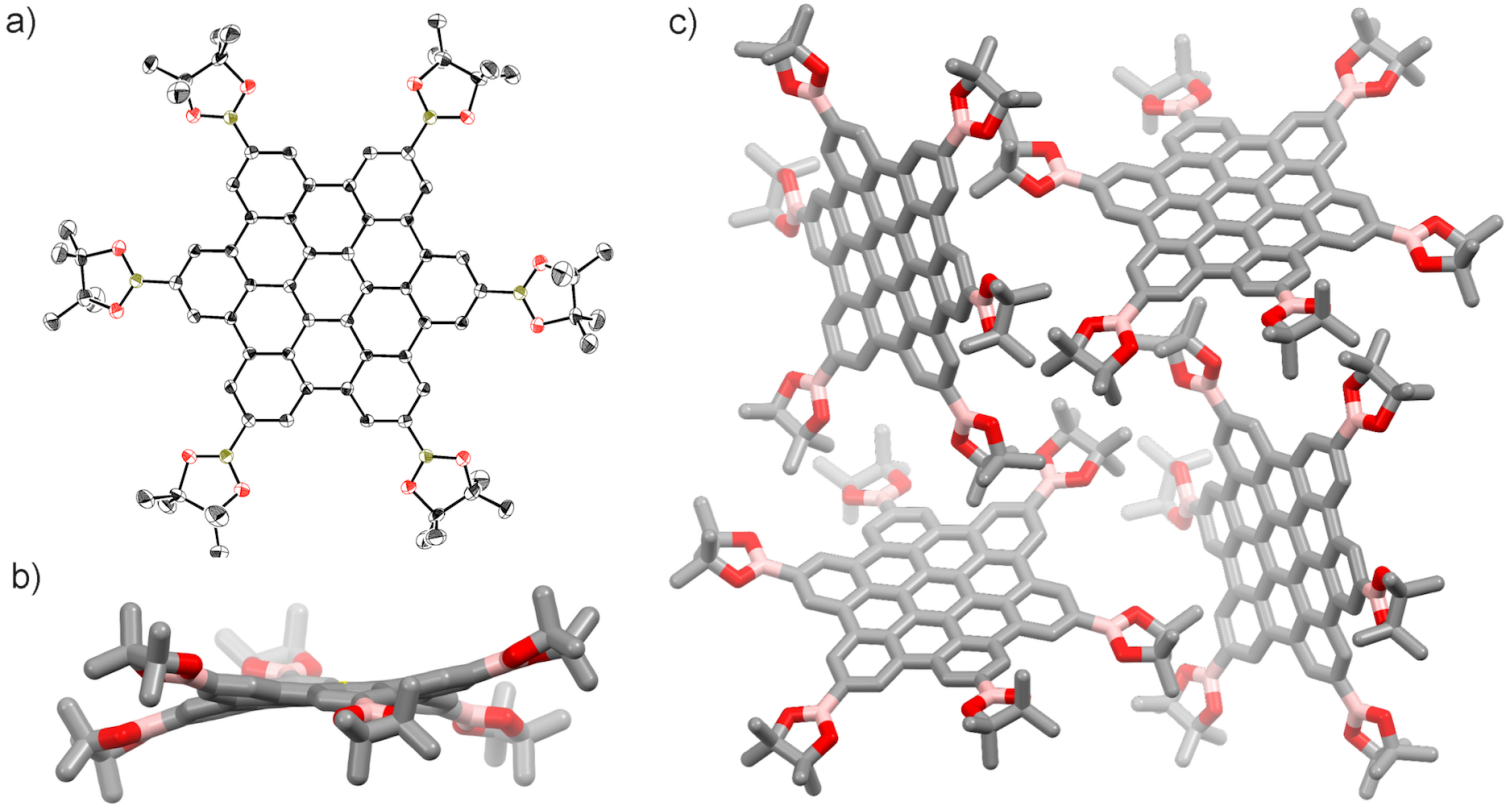
The structure of **1** confirmed by X-ray crystallographic analysis. (a) ORTEP drawing of **1** with thermal ellipsoids set to 50% probability; all hydrogen atoms and solvent molecules (pentane) are omitted for clarity; gray: carbon; olive green: boron; red: oxygen. (b) Side view of **1**. (c) Packing mode of **1**.

The hexaborylated HBC **1** was subjected to X-ray crystallographic analysis. Orange single crystals were formed by the diffusion of pentane vapor to a 1,1,1-trichloroethane solution of **1** synthesized by the C–H borylation method. The crystal structure confirmed that the C–H borylation proceeded regioselectively at the least hindered C–H bonds of HBC (Figure 3a). An equivalent amount of pentane for **1** was co-crystallized. As illustrated in Figure 3b and 3c, **1** is slightly distorted to have *S*_6_ symmetry, and no π–π interaction is observed in the packing mode reflecting the steric hindrance of boryl groups. This result corresponds with the high solubility of **1**.

**Figure 4:**
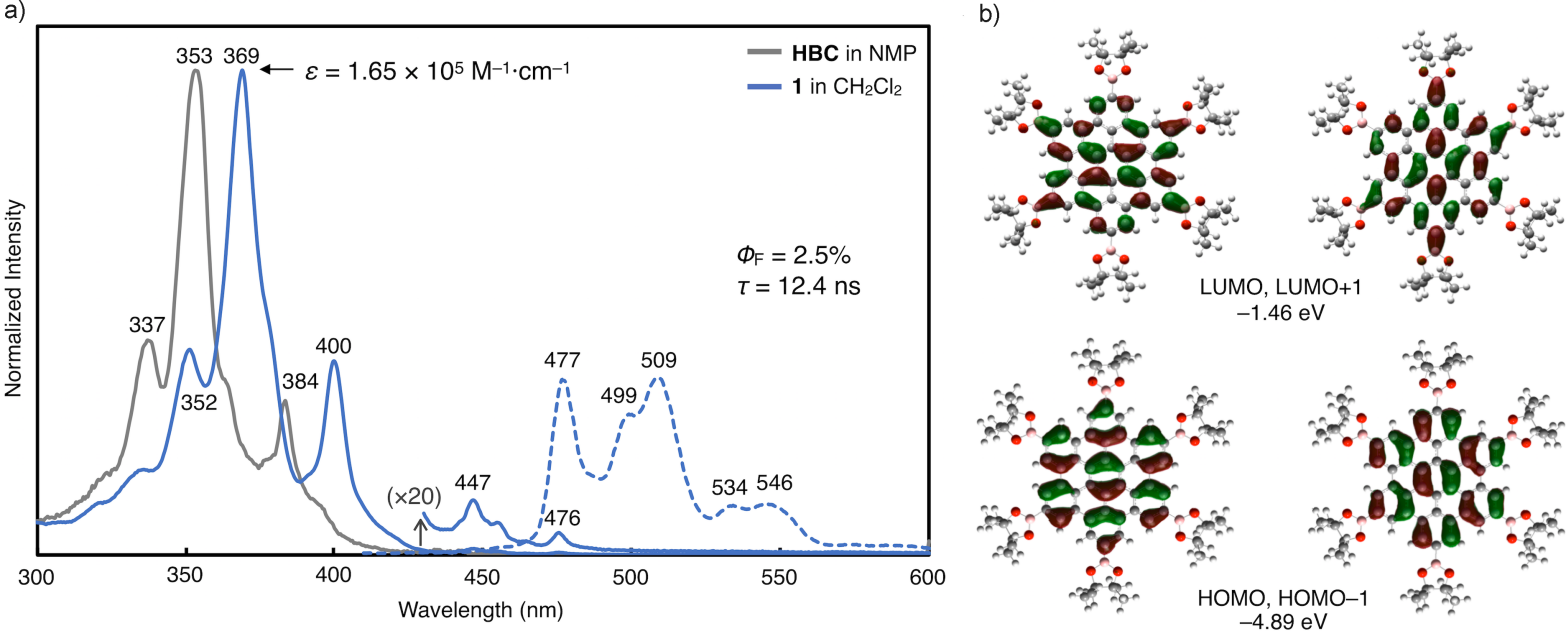
Photophysical properties of **1**. (a) UV–vis absorption (solid lines) spectra, fluorescence (dotted line) spectrum, absolute fluorescence quantum yield (Φ_F_) and fluorescence lifetime (τ). (b) Frontier molecular orbitals and energy levels of **1** calculated at the B3LYP/6-31G(d) level of theory.

In order to analyze the optoelectronic properties of **1**, the UV–vis absorption spectra, fluorescence spectrum, absolute fluorescence quantum yield and fluorescence lifetime were measured (Figure 4a and Figure S1, Supporting Information File 1). In comparison with the absorption spectra of pristine HBC, the characteristic three peaks of the HBC core were red-shifted about 15 nm for **1**. The molar absorption coefficient (ε) at the maximum absorption wavelength of **1** (369 nm) was 1.65 × 10^5^ M^−1^·cm^−1^. The maximum absorption peak and the minimum fluorescence peak were nearly overlapped, which indicated that these peaks can be assigned to 0–0 transitions. The absolute fluorescence quantum yield (Φ_F_) and fluorescence lifetime (τ) were 2.5% and 12.4 ns, respectively. According to the equations Φ_F_ = *k*_r_ × τ and *k*_r_ + *k*_nr_ = τ^−1^, the radiative (*k*_r_) and nonradiative (*k*_nr_) decay rate constants from the singlet excited state were determined (*k*_r_ = 2.0 × 10^6^ s^−1^; *k*_nr_ = 7.9 × 10^7^ s^−1^). The frontier molecular orbitals of **1** calculated by DFT calculations at the B3LYP/6-31G(d) level of theory are shown in Figure 4b. Owing to the highly symmetrical structure of **1**, both its HOMO and HOMO−1 as well as its LUMO and LUMO+1 are degenerate. The HOMO and LUMO energies of **1** increase under the influence of the σ-donation of the boryl groups, resulting in a decrease of the HOMO–LUMO energy gap. This is in line with the bathochromic shift observed in the absorption spectrum of **1** from that of HBC.

## Conclusion

In summary, the six-fold C–H borylation of unsubstituted HBC has been achieved. The optimal conditions were discovered through the screening of solvents, and **1** was obtained in 27% yield. The isolated yield of 0.8% in a stepwise method via a Miyaura–Ishiyama borylation indicates the advantage of the present method. The analyzed structure from X-ray crystallography confirmed the regioselective C–H borylations at the least sterically hindered C–H bonds of HBC. Optoelectronic measurements of **1** revealed bathochromic shifts of the absorption bands compared with unsubstituted HBC. DFT calculations also showed that the boryl groups increased the energies of frontier molecular orbitals and decreased the HOMO–LUMO energy gap. Compound **1** will contribute to a step-economical synthesis of various HBC derivatives as a platform, and furthermore, the currently developed borylation method gives insight into the functionalization of a variety of large and poorly soluble PAHs.

## Experimental

### General

Unless otherwise noted, all materials including dry solvent were obtained from commercial suppliers and used without further purification. Work-up and purification procedures were carried out with reagent-grade solvents under air. HBC (sublimated) was purchased from FUJIFILM Wako Pure Chemical Corporation (Catalog No. W01N40HZ370). Compound **2** was synthesized according to a reported procedure [23]. Flash column chromatography was performed with KANTO Silica Gel 60N (spherical, neutral, 40–100 µm). Preparative recycling gel permeation chromatography (GPC) was performed with a JAI LaboACE LC-5060 instrument equipped with JAIGEL-2H columns using chloroform as an eluent. Retsch MM400, zirconia milling jars and zirconia balls were used for pulverizing at 30 Hz and 60 min. The high-resolution mass spectrum (HRMS) was obtained from a JEOL JMS-S3000 SpiralTOF (MALDI–TOF MS). Nuclear magnetic resonance (NMR) spectra were recorded on a JEOL ECS-600 (^1^H 600 MHz, ^13^C 150 MHz) spectrometer or a JEOL ECA 600II spectrometer with UltraCool^TM^ probe (^1^H 600 MHz, ^13^C 150 MHz). Chemical shifts for ^1^H NMR are expressed in parts per million (ppm) relative to CHCl_3_ (δ 7.26 ppm). Chemical shifts for ^13^C NMR are expressed in ppm relative to CDCl_3_ (δ 77.0 ppm). Data are reported as follows: chemical shift, multiplicity (s = singlet) and integration.

### General procedure of the C–H borylation of HBC

To a 20-mL J-Young^®^ Schlenk tube equipped with a magnetic stirring bar was added HBC (10.0 mg, 19.1 μmol, 1.0 equiv). The flask was put in an argon-filled glove box, and a solution of [Ir(OMe)cod]_2_ (1.9 mg, 2.9 μmol, 15 mol %) and 3,4,7,8-tetramethyl-1,10-phenanthroline (tmphen; 1.4 mg, 5.9 μmol, 31 mol %) in a dry solvent (300 μL) was added to the flask. Then 4,4,5,5-tetramethyl-1,3,2-dioxaborolane (HBpin; 44.1 mg, 344 μmol, 18 equiv) was added to the mixture and the Schlenk tube was sealed with a J-Young^®^ screw tap. The resultant mixture was stirred at 80 °C for 2 days. After cooling the mixture to room temperature, the reaction mixture was passed through short-path silica gel with chloroform. The solvent was removed under reduced pressure. ^1^H NMR yields were determined by using 2,3,4-trimethoxybenzaldehyde as an internal standard in CDCl_3_. The crude material obtained from the reaction with CPME was purified by GPC (eluent: chloroform) to afford **1** (6.7 mg, 27%). ^1^H NMR (600 MHz, CDCl_3_) δ 9.78 (s, 1H), 1.61 (s, 6H).

### Stepwise synthesis of **1** from **3**

The Scholl reaction of **2** was performed according to a literature procedure [19]. To a 1 L two-necked round bottom flask containing a magnetic stirring bar were added dichloromethane (400 mL) and compound **2** (998 mg, 773 µmol) under nitrogen atmosphere. Then a solution of anhydrous FeCl_3_ (4.02 g, 24.8 mmol, 32 equiv) in nitromethane (10 mL) was added via syringe. The mixture was stirred at room temperature for 21 h. After quenched by methanol, the precipitate was collected and washed by dichloromethane, methanol and acetone. The reaction mixture containing **3** was obtained as a brown solid, which was used for the next reaction without further purifications.

A 15 mL Schlenk tube containing a magnetic stirring bar and KOAc (48.0 mg, 0.489 mmol, 6.2 equiv) was dried by a heat gun, cooled to room temperature and then refilled with nitrogen. To this vessel were added **3** (100 mg, 78.3 μmol, 1.0 equiv), Pd(dppf)Cl_2_ (28.6 mg, 39.1 μmol, 0.5 equiv), bis(pinacolato)diboron (B_2_pin_2_; 199 mg, 783 μmol, 10 equiv), toluene (0.36 mL) and *N,N*-dimethylformamide (DMF; 0.36 mL). The mixture was stirred at 80 °C for 18 h. After cooling the mixture to room temperature, the reaction mixture was passed through short-path silica gel with chloroform. The solvent was removed under reduced pressure. The crude material was purified by GPC (eluent: chloroform) and recrystallized with 1,1,1-trichloroethane and pentane to furnish **1** (1.1 mg, 0.8% yield based on **2**) as an orange solid. ^1^H NMR (600 MHz, CDCl_3_) δ 9.78 (s, 1H), 1.61 (s, 6H); ^13^C NMR (150 MHz, CDCl_3_) δ 25.6, 84.4, 122.4, 127.0, 127.3, 128.8, 129.9; HRMS (MALDI–TOF MS) *m/z*: [M]^+^ calcd for C_78_H_84_B_6_O_12_, 1278.6571; found, 1278.6582.

### X-ray crystallography

Details of the crystal data and a summary of the intensity data collection parameters for **1** are listed below. A suitable crystal was mounted with mineral oil on a MiTeGen MicroMounts and transferred to the goniometer of the kappa goniometer of a RIGAKU XtaLAB Synergy-S system with 1.2 kW MicroMax-007HF microfocus rotating anode (Graphite-monochromated Mo Kα radiation (λ = 0.71073 Å)) and PILATUS200K hybrid photon-counting detector. Cell parameters were determined and refined, and raw frame data were integrated using CrysAlis^Pro^ (Agilent Technologies, 2010). The structures were solved by direct methods with (SHELXT) [24] and refined by full-matrix least-squares techniques against *F*^2^ (SHELXL-2018/3) [25] by using Olex2 software package [26]. The intensities were corrected for Lorentz and polarization effects. Non-hydrogen atoms were refined anisotropically, and hydrogen atoms were placed using AFIX instructions.

Crystal Data for **1**: CCDC 1981141. C_88_H_108_B_6_O_12_ (C_78_H_84_B_6_O_12_·2C_5_H_12_) (*M* =1422.60 g·mol^–1^), crystal size: 0.2 × 0.2 × 0.2 mm, cubic, space group *Pa*-3 (no. 205), *a* = 19.9143(8) Å, *V* = 7897.6(10) Å^3^, *Z* = 4, *T* = 123(2) K, μ(Mo Kα) = 0.076 mm^−1^, *D*_calc_ = 1.196 g/cm^3^, 8158 reflections measured (4.09° ≤ 2θ ≤ 55.862°), 2594 unique (*R*_int_ = 0.0529). Goodness-of-fit on *F*^2^: 1.093. *R*_1_ = 0.0733, *wR*_2_ = 0.2041 (*I* > 2*σ*(*I*)) and *R*_1_ = 0.1252, *wR*_2_ = 0.2378 (all data).

### Photophysical measurements

UV–vis absorption spectra were recorded on a Shimadzu UV-3510 spectrometer with a resolution of 0.5 nm. The emission spectrum was measured on a Shimadzu RF-6000 spectrometer with a resolution of 0.5 nm. Absolute fluorescence quantum yield (Φ_F_) was determined on a Shimadzu RF-6000 spectrometer using a calibrated integrating sphere system upon excitation at 340 nm. For FL lifetime measurement, a Hamamatsu Photonics Quantaurus-Tau^®^ fluorescence lifetime spectrometer C11367-21 with LED as a light source was used (excitation: 365 nm, emission: 510 nm, frequency: 1 MHz). Dilute solutions in NMP or spectral grade dichloromethane in a 1 cm square quartz cell were used for measurements.

### Computational study

The Gaussian 16 program [27] running on a NEC LX 110Rh system was used for optimization (B3LYP/6-31G(d)) [28–29]. Structures were optimized without any symmetry assumptions.

## Supporting Information

Supporting information features copies of ^1^H and ^13^C NMR spectra and UV-vis absorption spectra of compound **1** and Cartesian coordinates of the optimized structure of **1**.

File 1 UV–vis spectra, NMR spectra and Cartesian coordinates.

File 2 Crystallographic information file of compound **1**.
