# Supplementary material for: Six-fold C–H borylation of hexa-peri-hexabenzocoronene
Source: Beilstein J Org Chem. 2020 Mar 13;16:391–7. doi: 10.3762/bjoc.16.37 (PMC7082694; doi:10.3762/bjoc.16.37)
Supplement: File 1 — UV–vis spectra, NMR spectra and Cartesian coordinates. [file Beilstein_J_Org_Chem-16-391-s001.pdf]

BEILSTEIN JOURNAL OF ORGANIC CHEMISTRY

Supporting Information

for

Six-fold C–H borylation of hexa-*peri*-hexabenzocoronene

Mai Nagase, Kenta Kato, Akiko Yagi, Yasutomo Segawa and Kenichiro Itami

Beilstein J. Org. Chem. **2020,** *16,* 391–397. doi:10.3762/bjoc.16.37

UV–vis spectra, NMR spectra and Cartesian coordinates

License and Terms: This is a supporting information file under the terms of the Creative Commons Attribution License (https://creativecommons.org/licenses/by/4.0). Please note that the reuse, redistribution and reproduction in particular requires that the authors and source are credited.
The license is subject to the *Beilstein Journal of Organic Chemistry* terms and conditions: (https://www.beilstein-journals.org/bjoc)

1. Photophysical measurements.

UV-vis absorption spectra of **1** in CH_2Cl_2 and NMP are shown in Figure S1. It can be concluded that the bathochromic shift of **1** compared with HBC is not due to the solvent effect of NMP.

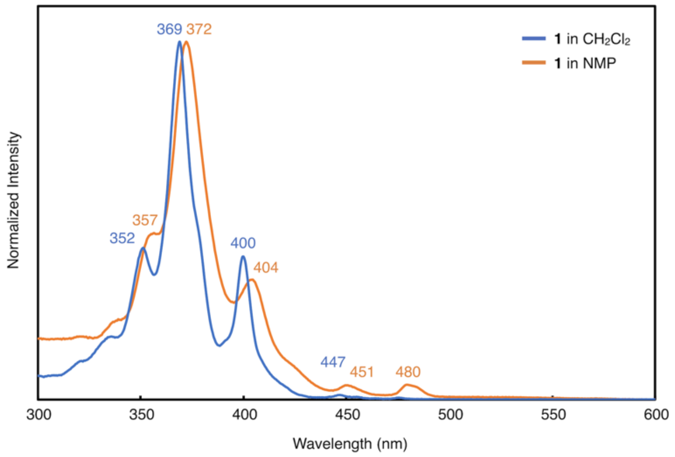

Figure S1: UV-vis absorption spectra of **1** in CH_2Cl_2 and NMP.

2. ^{1}H NMR and ^{13}C NMR spectra of 1.

^{1}H NMR spectrum of **1** obtained from C–H borylation (600 MHz, $CDCl_3$)

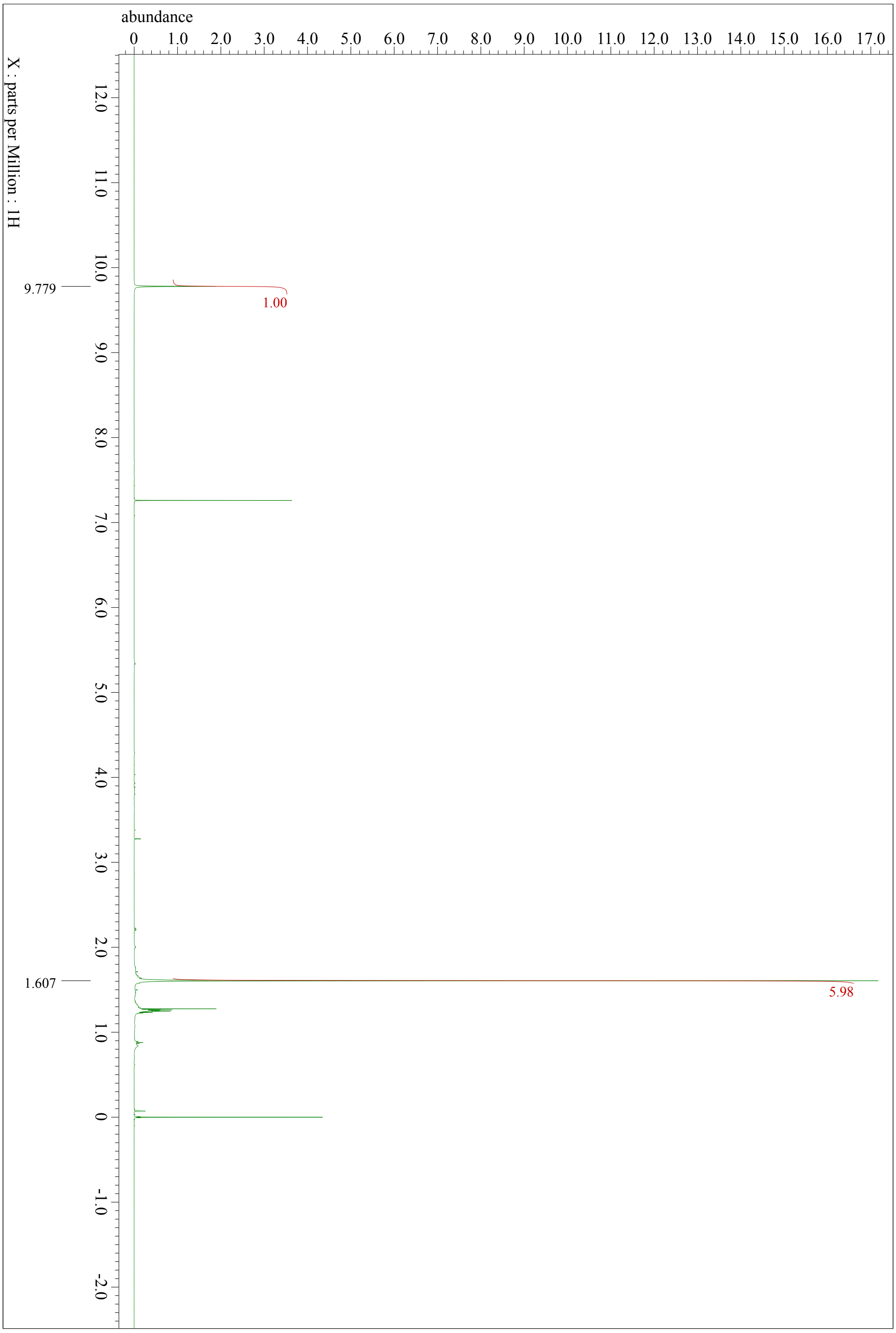

^{1}H NMR spectrum of **1** obtained from the stepwise synthesis (600 MHz, $CDCl_3$)

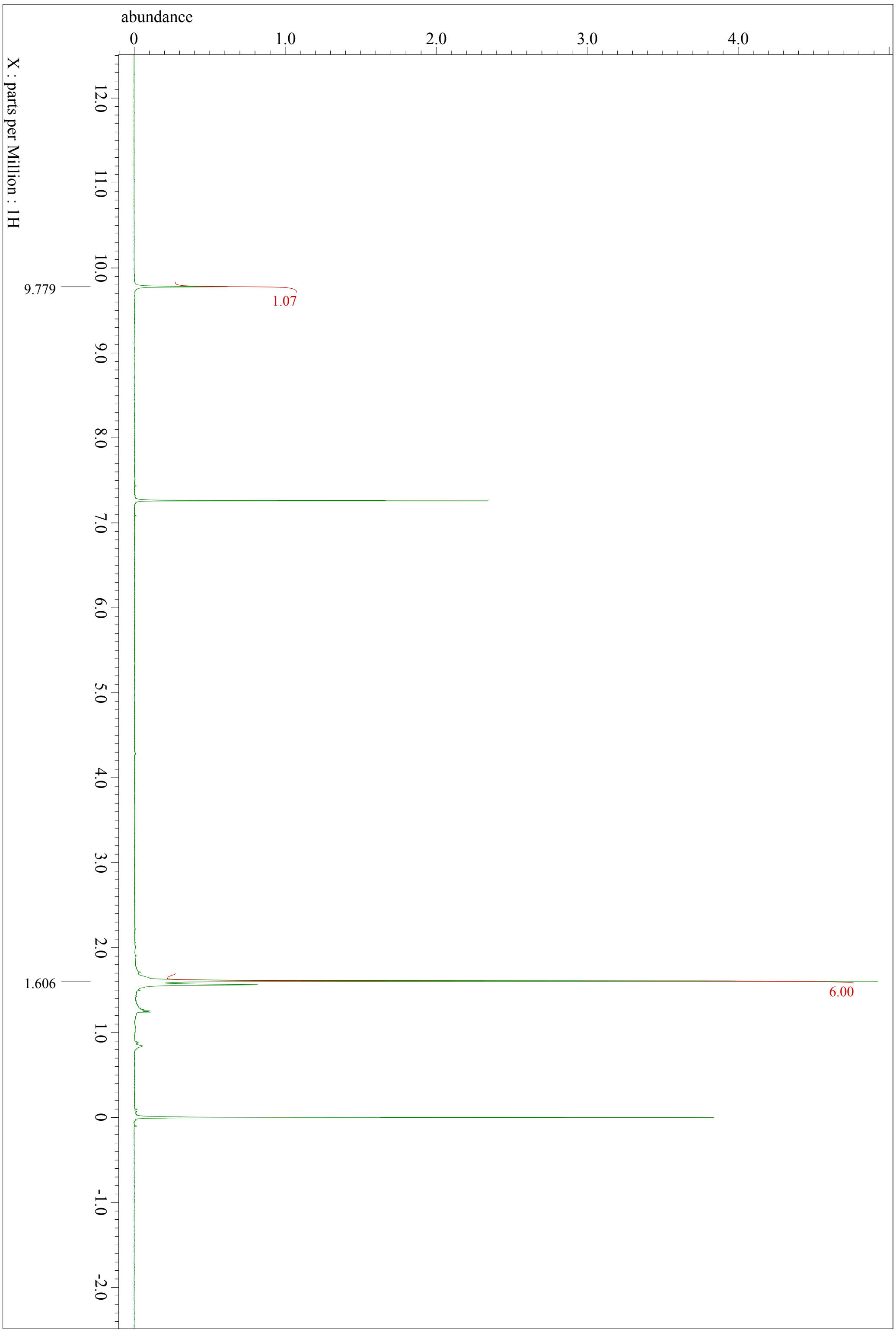

^{13}C NMR spectrum of **1** (150 MHz, $CDCl_3$)

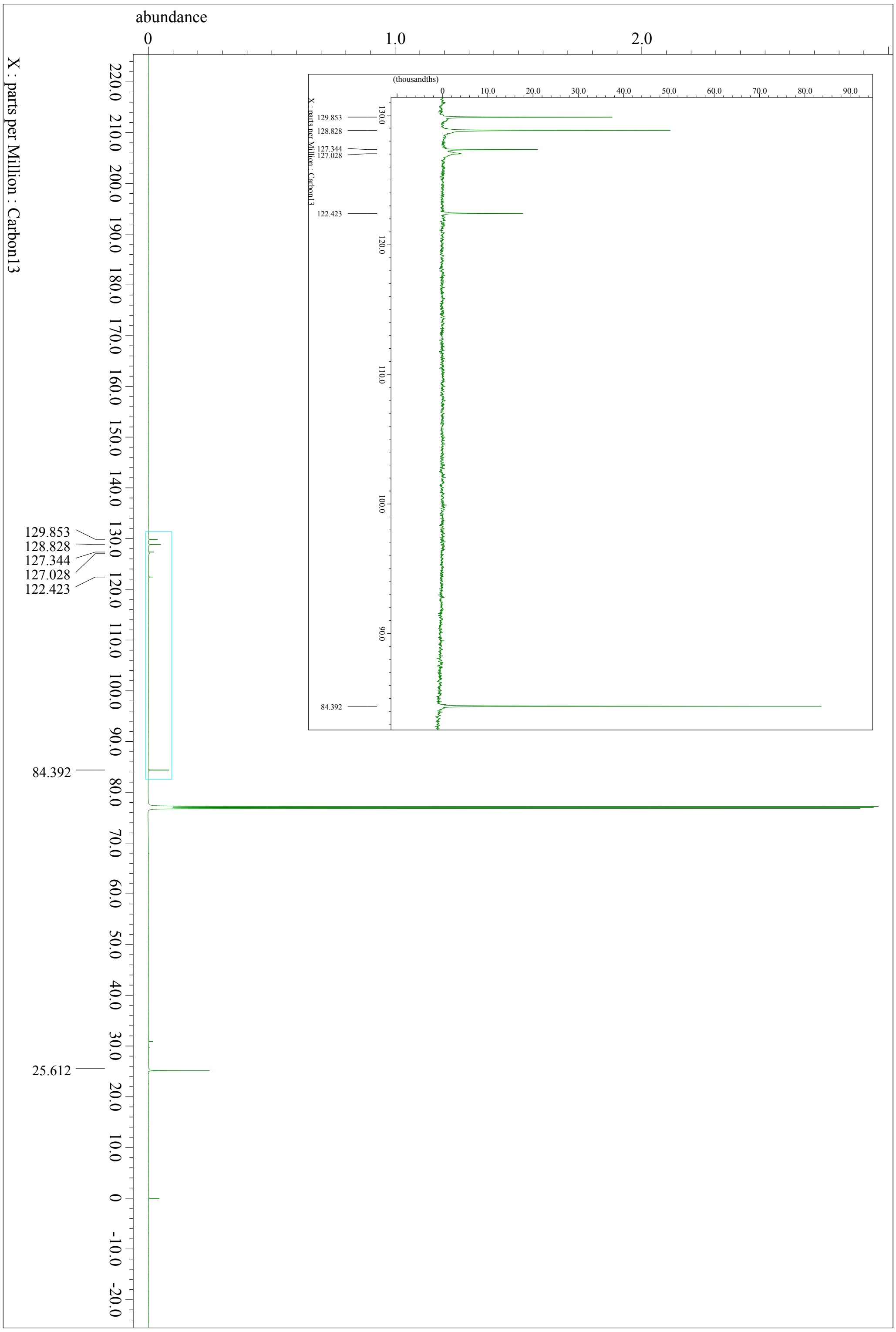

3. Cartesian coordinates of the optimized structure of 1.

O	-6.674598	-2.906313	3.560513	O	-3.560538	-6.674575	-2.906335	O	3.560538	6.674575	2.906335
O	-6.367913	-1.065752	4.893645	O	-4.893645	-6.367913	-1.065756	O	4.893645	6.367913	1.065756
C	-2.303582	-0.742956	1.545209	C	-1.545218	-2.303574	-0.742965	C	1.545218	2.303574	0.742965
C	-3.083331	-1.876932	1.175943	C	-1.175959	-3.083316	-1.876948	C	1.175959	3.083316	1.876948
C	-2.685913	0.024986	2.682908	C	-2.682911	-2.685909	0.024983	C	2.682911	2.685909	-0.024983
C	-4.224602	-2.190997	1.924314	C	-1.924338	-4.224579	-2.191021	C	1.924338	4.224579	2.191021
H	-4.840694	-3.037892	1.649190	H	-1.649232	-4.840656	-3.037932	H	1.649232	4.840656	3.037932
C	-4.620092	-1.436348	3.031293	C	-3.031306	-4.620080	-1.436360	C	3.031306	4.620080	1.436360
C	-3.835779	-0.338982	3.394596	C	-3.394595	-3.835780	-0.338979	C	3.394595	3.835780	0.338979
H	-4.151090	0.240041	4.253562	H	-4.253543	-4.151106	0.240061	H	4.253543	4.151106	-0.240061
C	-7.885233	-2.798915	4.360644	C	-4.360667	-7.885211	-2.798943	C	4.360667	7.885211	2.798943
B	-5.896990	-1.805765	3.834613	B	-3.834625	-5.896976	-1.805778	B	3.834625	5.896976	1.805778
C	-1.139858	-0.371266	0.769195	C	-0.769200	-1.139854	-0.371270	C	0.769200	1.139854	0.371270
C	-6.805191	-2.560190	6.717848	C	-6.717875	-6.805210	-2.560170	C	6.717875	6.805210	2.560170
H	-6.028742	-3.259777	6.392776	H	-6.392830	-6.028751	-3.259757	H	6.392830	6.028751	3.259757
H	-7.553960	-3.114440	7.293672	H	-7.293689	-7.553989	-3.114416	H	7.293689	7.553989	3.114416
H	-6.338781	-1.822805	7.378460	H	-7.378487	-6.338818	-1.822774	H	7.378487	6.338818	1.822774
C	-8.296256	-4.201607	4.804422	C	-4.804473	-8.296208	-4.201633	C	4.804473	8.296208	4.201633
H	-8.554416	-4.802428	3.926441	H	-3.926500	-8.554341	-4.802477	H	3.926500	8.554341	4.802477
H	-9.174810	-4.162967	5.458767	H	-5.458804	-9.174772	-4.162999	H	5.458804	9.174772	4.162999
H	-7.488188	-4.710985	5.334097	H	-5.334171	-7.488136	-4.710982	H	5.334171	7.488136	4.710982
C	-7.434231	-1.829446	5.523642	C	-5.523645	-7.434231	-1.829445	C	5.523645	7.434231	1.829445
C	-8.504049	-0.853480	6.009816	C	-6.009781	-8.504065	-0.853479	C	6.009781	8.504065	0.853479
H	-8.082796	-0.204616	6.784419	H	-6.784362	-8.082819	-0.204583	H	6.784362	8.082819	0.204583
H	-9.355368	-1.390525	6.443865	H	-6.443848	-9.355378	-1.390520	H	6.443848	9.355378	1.390520
H	-8.868361	-0.217122	5.200296	H	-5.200239	-8.868385	-0.217153	H	5.200239	8.868385	0.217153
C	-8.963084	-2.197592	3.448309	C	-3.448330	-8.963081	-2.197650	C	3.448330	8.963081	2.197650
H	-8.685205	-1.194052	3.111152	H	-3.111156	-8.685221	-1.194110	H	3.111156	8.685221	1.194110
H	-9.933663	-2.139000	3.952258	H	-3.952290	-9.933654	-2.139065	H	3.952290	9.933654	2.139065
H	-9.071990	-2.831920	2.563183	H	-2.563216	-9.071987	-2.831993	H	2.563216	9.071987	2.831993
O	-2.906329	3.560531	6.674582	O	2.906329	-3.560531	-6.674582	O	6.674598	2.906313	-3.560513
O	-1.065752	4.893640	6.367918	O	1.065752	-4.893640	-6.367918	O	6.367913	1.065752	-4.893645
C	-0.742953	1.545206	2.303586	C	0.742953	-1.545206	-2.303586	C	2.303582	0.742956	-1.545209
C	-1.876936	1.175946	3.083328	C	1.876936	-1.175946	-3.083328	C	3.083331	1.876932	-1.175943
C	0.024996	2.682898	2.685923	C	-0.024996	-2.682898	-2.685923	C	2.685913	-0.024986	-2.682908
C	-2.191009	1.924326	4.224591	C	2.191009	-1.924326	-4.224591	C	4.224602	2.190997	-1.924314
H	-3.037921	1.649221	4.840667	H	3.037921	-1.649221	-4.840667	H	4.840694	3.037892	-1.649190
C	-1.436350	3.031296	4.620090	C	1.436350	-3.031296	-4.620090	C	4.620092	1.436348	-3.031293
C	-0.338970	3.394585	3.835790	C	0.338970	-3.394585	-3.835790	C	3.835779	0.338982	-3.394596
H	0.240064	4.253539	4.151112	H	-0.240064	-4.253539	-4.151112	H	4.151090	-0.240041	-4.253562
C	-2.798938	4.360661	7.885218	C	2.798938	-4.360661	-7.885218	C	7.885233	2.798915	-4.360644
B	-1.805771	3.834617	5.896984	B	1.805771	-3.834617	-5.896984	B	5.896990	1.805765	-3.834613
C	-0.371263	0.769192	1.139861	C	0.371263	-0.769192	-1.139861	C	1.139858	0.371266	-0.769195
C	-2.560170	6.717867	6.805208	C	2.560170	-6.717867	-6.805208	C	6.805191	2.560190	-6.717848
H	-3.259756	6.392816	6.028750	H	3.259756	-6.392816	-6.028750	H	6.028742	3.259777	-6.392776
H	-3.114418	7.293684	7.553984	H	3.114418	-7.293684	-7.553984	H	7.553960	3.114440	-7.293672
H	-1.822774	7.378478	6.338814	H	1.822774	-7.378478	-6.338814	H	6.338781	1.822805	-7.378460
C	-4.201628	4.804463	8.296217	C	4.201628	-4.804463	-8.296217	C	8.296256	4.201607	-4.804422
H	-4.802469	3.926489	8.554355	H	4.802469	-3.926489	-8.554355	H	8.554416	4.802428	-3.926441
H	-4.162994	5.458798	9.174778	H	4.162994	-5.458798	-9.174778	H	9.174810	4.162967	-5.458767
H	-4.710981	5.334156	7.488144	H	4.710981	-5.334156	-7.488144	H	7.488188	4.710985	-5.334097
C	-1.829443	5.523641	7.434235	C	1.829443	-5.523641	-7.434235	C	7.434231	1.829446	-5.523642
C	-0.853479	6.009784	8.504067	C	0.853479	-6.009784	-8.504067	C	8.504049	0.853480	-6.009816
H	-0.204587	6.784368	8.082821	H	0.204587	-6.784368	-8.082821	H	8.082796	0.204616	-6.784419
H	-1.390522	6.443849	9.355380	H	1.390522	-6.443849	-9.355380	H	9.355368	1.390525	-6.443865
H	-0.217148	5.200246	8.868388	H	0.217148	-5.200246	-8.868388	H	8.868361	0.217122	-5.200296
C	-2.197641	3.448325	8.963086	C	2.197641	-3.448325	-8.963086	C	8.963084	2.197592	-3.448309
H	-1.194101	3.111153	8.685225	H	1.194101	-3.111153	-8.685225	H	8.685205	1.194052	-3.111152
H	-2.139057	3.952284	9.933660	H	2.139057	-3.952284	-9.933660	H	9.933663	2.139000	-3.952258
H	-2.831982	2.563209	9.071992	H	2.831982	-2.563209	-9.071992	H	9.071990	2.831920	-2.563183
